# A global review of racial, ethnic and socio-economic disparities in multisystem inflammatory syndrome in children related to COVID-19

**DOI:** 10.3389/fpubh.2022.996311

**Published:** 2022-10-20

**Authors:** Zoha Asghar, Kanza Sharaf, Faran Ahmed Butt, Omer Ahmed Shaikh, Manahil Shekha, Abdul Waris, Irfan Ullah, Abdulqadir J. Nashwan

**Affiliations:** ^1^Department of Medicine, Ziauddin University, Karachi, Pakistan; ^2^Kabir Medical College, Gandhara University, Peshawar, Pakistan; ^3^Institute of Public Health and Social Science (IPH&SS), Khyber Medical University, Peshawar, Pakistan; ^4^Hamad Medical Corporation, Doha, Qatar

**Keywords:** multi-system inflammatory syndrome, COVID-19, SARS CoV-2, children, infant, pediatric multi-system inflammatory syndrome, Kawasaki disease, health disparities

## Abstract

With over 500 million confirmed cases and 6.2 million deaths worldwide, the novel coronavirus has highlighted the underlying disparities in healthcare, unpreparedness to deal with a new disease and the need for monitoring and surveillance for a post-infectious syndrome as well as complicated diseases. Initially, children were thought to be spared but reports of a new phenomenon manifesting as Kawasaki-like disease, toxic shock syndrome, and multi-system inflammatory syndrome, which developed after a few weeks of severe COVID-19 infection, emerged in the pediatric population. As the pandemic progressed, increased prevalence of multi-system inflammatory syndrome in children (MIS-C) related to COVID-19 was seen in non-Hispanic blacks, Asians, and Latinos as compared to the white population drawing attention to a possible role of ethnicity and socio-economic disparities. The CDC currently reports that 31% of MIS-C cases were seen in Black Non-Hispanics and 26% in Latinos, who were historically more affected in previous pandemics. Furthermore, MIS-C cases in developing countries showed higher mortality as compared to high-income countries, which points toward the role of social determinants of health and limitations in a low-resource set up in increasing the disease burden of MIS-C, which should be treated as a public health emergency. Our review highlights the role of ethnicity, socio-economic factors, comorbidities, and differences in populations affected by MIS-C in high-income vs. low- and middle-income countries.

## Introduction

What started as a mere flu in December 2020, the Coronavirus (COVID-19) pandemic caused by the SARS-CoV-2 virus resulted in over 6.2 million deaths from nearly 500 million confirmed cases (1). Early reports showed that children were mostly spared from severe forms of illnesses associated with COVID-19, with only 2% of cases diagnosed in the pediatric population up till February 2020 (2). Epidemiological studies further suggested that compared with adult patients' the clinical manifestations of COVID-19 in children were mostly mild and showed minimum mortality (3). However, this judgment was revised in April 2020 when several countries in Europe and North America reported cases of young patients with “Multi-System Inflammatory Syndrome in Children (MIS-C)” associated with SARS-CoV-2 also known as Kawasaki-like syndrome and toxic shock syndrome (4). Reports of clusters of children and adolescents affected by MIS-C admitted to ICU or requiring mechanical ventilation emerged from the UK, Italy, and New York, followed by other parts of the US (5, 6). It was further found that Black and Hispanic children formed an overwhelming majority (66%) of those who developed the life-threatening MIS-C (7).

Although rare, this condition needs extensive surveillance in areas with a high burden of COVID-19, which have shown consistent patterns of racial/ethnic differences (8, 9). The exact role of race and ethnicity on clinical outcomes of COVID-19 is unknown, but given the general consensus, there is a greater need to examine the factors behind disproportionate levels of adverse clinical outcomes (10). Particularly in pediatric populations where reports of severe disease have been limited owing to incomplete public health data worldwide (11). The current studies lack data on race and ethnicity-specific presentations of the syndrome, the mechanism of genetic predisposition to MIS-C, and further research into its' worldwide distribution, given that it should be treated as a public health emergency that requires intensive care and surveillance. Literature regarding disparities in COVID-19 has largely addressed the adult population while the extent of racial and ethnic disparities in children is relatively unknown. While MIS-C initially emerged in the US and Europe, soon after cases were reported in the developing countries which was a source of immediate concern and attention for communities worldwide. It is also imperative to understand the variation in clinical features and severity of this disease in affected countries and to assess the potential role played by social determinants of health. Given the scarcity of data in the current literature, in this review we compare the distribution of MIS-C in High-Income Countries (HICs) and Low-Income Countries (LMICs) and explore the role of social and living conditions, comorbidities, and ethnicity in the development of MIS-C as well as the extent of severe forms of the disease in certain populations.

### Case definition

Variously termed as Kawasaki-like disease, pediatric multi system inflammatory syndrome temporally associated with COVIRD-19 (PIMS-TS) or MIS-C, case definitions have been produced by the World Health Organization, US Centers for Disease Control and Prevention and the UK Royal College of pediatrics (Table 1).

**Table 1:** Case definitions by WHO, US-CDC and Royal College of Pediatrics, UK.

**World Health Organization (WHO) (15th May 2020) Multi-system inflammatory syndrome in children and adolescents temporally related to COVID-19**	**US-Center of Disease Control (CDC) (14th May 2020) Multi-system inflammatory syndrome in children (MIS-C)**	**Royal College of Pediatrics and Child Health (RCPCH) (1st May 2020) Pediatric multisystem inflammatory syndrome temporally associated with COVID-19**
Fever >3 days AND elevated markers of inflammation (ESR, CRP or procalcitonin)	Fever ≥38.0°C for ≥24 hours or report of subjective fever lasting ≥ 24 h	Fever >38.5
0–19 years	< 21 years	Child
At least 2 of the following:		
1. Rash or bilateral non-purulent conjunctivitis or mucocutaneous inflammation signs (oral, hands or feet)	Evidence of clinically severe illness requiring hospitalization, with multisystem (>2) organ involvement (cardiac, renal respiratory, hematologic, gastrointestinal, dermatologic or neurological)	Persistent fever, inflammation (Neutrophilia, elevated CRP and lymphopenia) and evidence of single or multi-organ dysfunction (Shock, cardiac, respiratory, renal, gastrointestinal or neurological disorder) with additional features. This may include children fulfilling full or partial criteria for Kawasaki disease.
2. Hypotension or shock		
3. Features of myocardial dysfunction, pericarditis, valvulitis or coronary abnormalities (including ECHO findings or elevated Troponin/NT-proBNP)		
4. Evidence of coagulopathy (by PT, PTT, and elevated d-Dimers)		
5. Acute GI problems (diarrhea, vomiting or abdominal pain)		
No other obvious microbial cause of inflammation, including bacterial sepsis, staphylococcal/streptococcal shock syndromes	No alternative plausible diagnoses	Exclusion of any other microbial cause, including bacterial sepsis, staphylococcal or streptococcal shock syndromes, infections associated with myocarditis such as enterovirus (waiting for results of these investigations should not delay seeking expert advice).
Evidence of COVID-19 (RT-PCR, antigen test or serology positive), or likely contact with patients with COVID-19	Positive for current or recent SARS-CoV2 infection by RT PCR, serology or antigen test or COVID-19 exposure within the 4 weeks prior to onset of symptoms	SARS-CoV-2 PCR testing may be positive or negative

### Difference between Kawasaki like disease and MIS-C (STING PATHWAY)

In a retrospective observational study from Japan Kawasaki-disease Shock Syndrome (KDSS) and MIS-C were seen to overlap in clinical symptoms however are 2 separate entities. Kawasaki disease is a medium-sized vessel vasculitis usually in children under 5 years of age preceded by fever for at least 5 days and generalized inflammation that involves lymph nodes and particularly the skin and mucous membranes (12). KD progresses to Kawasaki disease shock syndrome (KDSS) when there is a 20% decrease in systolic blood pressure (13). COVID-19 can also develop severe course characterized by acute respiratory distress syndrome (ARDS) with a hyperinflammatory response (14). Multi-system inflammatory response in COVID-19 is characterized by systemic inflammation involving multiple organs such as cardiac, renal and gastrointestinal. Entry of a foreign antigen in the body causes activation of simulator of interferon genes (STING) which leads to release of inflammatory cytokines, predominantly type 1 IFN. The STING pathway is shown to be activated in KD brining about an inflammatory response that consists of neutrophils, macrophages and cytotoxic T cells which are seen on the histology of coronary arteries affected in KD (12). It has been reported that type 1 IFNs drive the immune response in SARS-CoV-2 (15). It was when Domizio et al. (16) identified a H-151 STING inhibitor that served as a therapeutic agent in reducing severe inflammation in SARS-CoV-2 an important role of STING pathway in COVID-19 was found. CT angiogram can also play an important role in young patients with symptoms of KD overlapping with COVID-19 to identify coronary aneurysms in a timely manner so that the fatal risk of thromboses and lumen narrowing can be diagnosed initially.

## Methods

In this narrative review, a thorough literature search of all peer-reviewed articles published between 31st December' 2019 to 1st April' 2022 was undertaken using keywords “multi-system inflammatory syndrome”, “COVID-19”, “SARS COV-2”, “coronavirus”, “children”, “infant”, “Kawasaki/Kawasaki-like disease”, “pediatrics”, “pediatric multi-system inflammatory syndrome” and any other relevant keywords and supplementary concepts were identified. Reference lists of the identified studies were also screened to look for similar studies. Multiple electronic databases were searched, which included PubMed, Google Scholar, Elsevier, Wiley Online Library, ScienceDirect, and WHO COVID-19 database. To increase the scope of our search, pre-prints from Medxriv were also included. An inclusion and exclusion criteria were pre-decided to guide our search, which is as follows:

**Table:** 

**Inclusion Criteria:**	**Exclusion Criteria:**
• Case reports, case series, cohort papers and case-control studies	• Opinions, letters, editorials, review articles
• Mean age ≤ 18 years	• Mean age > 18 years
• Addressing MIS-C as a complication or Kawasaki-like disease as a potential complication of SARS-CoV-2 • The country or region mentioned • Data on race and/or ethnicity mentioned • Addressing the socio-economic backgrounds of patients with MIS-C	• Studies that only discuss COVID-19 in the pediatric population but not MIS-C • Country or region not mentioned • Race and/or ethnicity are not mentioned • Articles not published in the English language
• Articles published in the English language	

### MIS-C and ethnicities

Previous studies published on COVID-19 in adults have highlighted racial/ethnic and socio-economic disparities and race discrimination continued in the provision of vaccines and treatments (17). A policy statement by American Academy of Pediatrics highlighted that the impact of racism starts from birth disparities which give rise to mental health problems and chronic stress conditions such as cortisol that predisposes children and adolescents to chronic disease (18). A study that enrolled 640 COVID-19 patients in the UK concluded that compared to white members, black individuals were at a 4 times higher risk of COVID-19, and it was twice higher in Asian and other non-white persons (19). However, there is limited data relevant to the pediatric population and if a certain ethnic group is at a higher risk of developing COVID-19, particularly MIS-C. Currently, the CDC website reports 7,880 MIS-C cases, 31% of which are Black Non-Hispanic and 26% are Hispanic/Latino, who are also disproportionately affected by COVID-19 (20). As seen in Kawasaki disease, which has shown a predominance in genetically susceptible children, similar patterns have been seen in MIS-C, in which higher prevalence was reported in Black, Hispanic, and South Asian populations (4, 21, 22). Dufort et al. reported a case series of 99 pediatric patients with confirmed and suspected MIS-C who belonged to New York. 31 out of 78 (40%) patients were black, and 31 out of 85 (36%) were Hispanic, compared to 29 out of 78 (37%) white patients (23). Adult deaths in New York showed a similar pattern. Compared to 22% African Americans and 29% Hispanic-Latinos in the overall population, the two groups accounted for 28 and 34% of deaths, respectively (24). Another study from New York reported that 45 and 39% of MIS-C patients were Hispanic/Latino and Black, respectively, compared to 9% White, 3% Asian, and 3% other ethnicities (25). In another cohort from NYC, among 223 patients meeting the MIS-C criteria, race/ethnicity data was available for 184 patients. 34.4% (75 patients) were Black, given the overall population of Black children 22.2 and 19.9% of patients under 20 years hospitalized due to COVID (26). From April to June 2020 the incidence of MIS-C in various states of America was 9.26, 8.92, and 2.94 times higher in Black, Hispanic or Latino, and Asians compared to white patients (27). Black and Hispanic populations also have the lowest rates of vaccination, and parents to date show hesitancy to vaccinate their children against COVID-19. Only 21% of children between 12 and 15 years were vaccinated, and 32% of those between 16 and 17 years of age among the racial/ethnic groups (28).

The racial disparities were not only confined to the United States of America (USA). Toubiana et al. reported 21 confirmed cases of Kawasaki Disease—like/MISC in the Paris region in France, where children of color were overrepresented, similar to what we saw in the USA. Twelve (57%) children had at least one parent from a sub-Saharan African or Caribbean Island, and 3 (14%) children were of Asian (Srilanka/China) descent (29). A study from the UK reported 15 cases of PIMS-TS (Pediatric Inflammatory Multisystem Syndrome-temporally associated with SARS-CoV-2) associated with COVID-19, and all children belonged to African/Afro-Caribbean, South Asian, mixed, or minority ethnic groups, which is relatively large given that only 3.3% of children are Black and 10% percent are Asian. In addition, these children showed severe cardiac symptoms, and 67% were admitted to the ICU (30). Another cluster of 8 children from the UK was reported with hyperinflammatory shock syndrome; all patients were Afro-Caribbean, Asian, and Middle Eastern, and all of them were admitted to the ICU and required mechanical ventilation, one child died, and the rest were discharged on surveillance (31). In Latin America, MISC was more widely reported compared to other developing countries, perhaps due to differing political opinions regarding the to approach the pandemic and lockdown policies making children more exposed to the infection during daily activities, which further support the point of view that children of Hispanic-Latino ethnicity are at a higher risk of developing MIS-C (32–34).

Table 2 summarizes the race and ethnicity findings of MIS-C.

**Table 2:** MIS-C according to Race and Ethnicity in different countries.

**Authors**	***N***	**Country/ Region**	**Race and ethnicities**
Jonat et al.	54	USA	Hispanic: 29.6%
			White: 35%
			African America: 19%
Abrams et al.	1,080	USA	Hispanic: 41%
			Non-Hispanic Black: 36%
			Non-Hispanic White: 14%
Shust et al.	8		Black and Hispanic affected disproportionately
Toubiana et al.	21	France	57% African ancestry and 14% Asian
Shelly Riphagen et al.	8	UK	6 Afro-Caribbean
			1 Asian
			1 Middle-Eastern
Kathleen Chiotos et al.	6	UK	2 Blacks
			2 Whites
			2 unknowns
			No Hispanic or Latino descent
Ramcharan et al.	15	UK	All patients were from:
			African/Afro-Caribbean
			South Asian
			Mixed
			Other minority ethnic groups
Masih et al.	1	UK	White Caucasian
Swann et al.	651	UK	Ethnicity was recorded in 88% (576/651) of cases:
			White: 57% (330/576)
			South Asian: 12% (67/576)
			Black: 10% (56/576)
			Children who met MISC criteria:
			White: 16
			Black: 9
			South Asian: 4
			Other: 16
			Missing: 7
Marisa Dolhnikoff et al.	1		African
Patrick Davies et al.	78	UK	Afro-Caribbean: 37
			Asian: 22
			White: 17
			Other: 2
Sussana Felsentein et al.	29	UK	Caucasian: 12 (41.4%)
			South East Asian: 6 (20.7%)
			East Asian: 2 (6.9%)
			African/Caribbean: 4 (13.8%)
			Unknown or multi-ethnic: 5 (17.2%)
Nele Alders et al.	57	UK	PIMS-TS patients were mostly of non-Caucasian ethnicity (*n* = 26 [84%] vs. *n* = 5 [50%])
Feldstein et al.	186	USA	35/186 (19%): White non-Hispanic
			46/186 (25%): Black non-Hispanic
			9/186 (5%): Another race and non-Hispanic
			57/186 (31%): Hispanic or Latino
			41/186 (22%): Unknown race
Dufort et al.	99	USA	Of 78 patients with data on race, 29 (37%) were white, 31 (40%) were black, 4 (5%) were Asian, and 14 (18%) were of other races; of 85 patients with data on ethnic group, 31 (36%) were Hispanic.
Kaushik et al.	33	USA	45%: Hispanic/Latino
			39%: Black
			9%: White
			3%: Asian
			3%: Others
Bandi et al.	474	USA	25.1%: non-Hispanic white
			43.2%: African American
			24.7%: Hispanic
			1.5%: Asian
			Remaining were identified as other races
Cheung et al.	17	USA	6 Jewish
			2 non-Hispanic
			4 Hispanic
			4 black
			1 Asian
Mariawy Riollano-Cruz	15	USA	10/15 patients (66%): Hispanic or Latino
			5/15 (34%): Other races
Shanana Godfred et al.	570	USA	40.5% Hispanic/Latino (Hispanic)
			33.1% non-Hispanic black (black) 13.2% non-Hispanic white (white)
Rivera-Figueroa et al.	1	USA	African American
Daniel et al.	1	USA	Multiracial (Caucasian and Hispanic)
Arnaldo Prata Barbosa et al.	79	Brazil	58% White
Kate Webb et al.	23	South Africa	Blacks (18)
			South African colored (5)
			No whites
Torres et al.	27	Chile	Latin American
Al-Aamria et al.	1	KSA	Arab

### MIS-C and comorbidities

The most common underlying condition in children with MIS-C was obesity (21, 23, 25, 26, 32, 35–38). The second most common comorbid highlighted in these cohorts was asthma (4, 22, 39–43). Hypothyroidism, non-alcoholic fatty liver disease, respiratory illness preceding 4 weeks of hospitalization, and glucose-6-phosphate-dehydrogenase deficiency were also seen in some cases (43, 44). Other comorbidities seen in severe COVID-19 cases were neurological problems, immunocompromised, premature births, and hematological problems, but only obesity was associated with MIS-C (40). Similar findings were seen in adult patients, where Black patients had higher prevalence of obesity, diabetes, hypertension, and chronic kidney disease compared to white patients (45). Black ethnicity was shown to be associated with comorbidities in a cohort where African American patients with 3 or more comorbidities formed a higher proportion of overall patients with severe COVID-19 (46). Particularly in the US, obesity was associated with factors such as age, race, Hispanic origin, and education of the household head which are directly related to ethnicity and one's socio-economic status (47). Furthermore, studies suggest that asthma is related to socio-economic factors, which are directly linked with ethnicity as well, such as environmental exposures, access to healthcare, stress, and psychological/cultural factors that have been associated with increased asthma morbidity (48).

### MIS-C in the developing world

Compared to High-Income Countries (HIC's), studies from the developing world have reported higher rates of hospitalization and deaths from MIS-C (49). The first case to be reported in South Asia was from Pakistan, where a cluster of 8 children reported confirmed MISC at a university hospital in the city of Lahore, all of whom showed cardiovascular involvement, and one died due to myocardial infarction and subsequent organ failure (50). Involvement of coronary artery disease and the overall infectivity rate in Pakistan in children younger than 20 years was higher (>10%) compared to the rest of the world (50, 51). In India, neonates and infants were affected by MIS-C with various manifestations ranging from in-utero exposure to SARS-CoV-2 in a premature infant (52), fatal respiratory distress syndrome with hypotensive shock and meningoencephalitis (53), cavitary lung lesions (54), persistent neutropenia (55) to dermatological involvement (56). In Iran, a retrospective study that covered 3 hospitals in regions most severely hit by the pandemic reported 45 confirmed cases of MISC and a mortality of 11% (*n* = 5) (57). Another case report from Iran showed a 5-year-old girl with Kawasaki disease like inflammatory syndrome with severe symptoms consistent with MISC that improved with standard treatment consisting of IVIG and anti-biotics (58).

At the time of writing this review, studies from Low- and Middle-Income Countries (LMIC's) showed a lesser number of MISC patients compared to HICs but a higher proportion of deaths (49). This is alarming due to a number of factors. Firstly, many physicians working on the front lines were stretched to not allocate enough time for clinical research and data collection. Secondly, lack of testing capacity overwhelmed in-patient facilities, and limited pediatric ICU and ventilator resources can cause many patients to return undiagnosed. Thirdly, children make up a large part of the population in LMICs compared to HICs and have more exposure to risk factors of lower respiratory diseases such as air pollution, incomplete immunization, malnutrition, greater prevalence of infectious diseases like TB and HIV, and overcrowded conditions with water and sanitation problems (59). Therefore, the number of cases of MISC can be largely underestimated.

It is essential to consider practical prevention strategies according to the limitations of populations in low-income countries. In communities with widespread transmission mass awareness and advocacy campaigns regarding the spread of the disease can be carried out with focus on limiting healthy children from visiting healthcare facilities, regular well-child visits for newborns and infants for preventive care and timely vaccinations, local availability of telephone triage system, immediate closure of schools and public places or at least restricting entrance for children as well as nutritional education for parents as diet plays a huge role in the development of immune system (60).

Table 3 summarizes findings from High-Income and Low-and Middle-Income countries based on the recent World Bank Classification (61).

**Table 3:** MISC in high income countries.

**Authors**	***N***	**Country/ Region**	**Clinical features**	**ICU admissions**	**Limitations**
Jonat et al.	54	USA	Mucocutaneous, GIT and neurologic symptom	57% with no deaths	
			Male−57%, Comorbid—Obesity		
Caro-Patón et al.	12	Spain	Cardiogenic shock, myocardial injury and ventricular dysfunction	100% with no death	Single center study
Elizabeth et al.	58	England	Vomiting (84%), abdominal pain (54%), diarrhea (52%), rash (52%), conjunctival injection (45%), female−57%	79% needed mechanical ventilation	
Lucio Verdoni et al.	Group 1: *n* = 19	Italy	Children showing immune response to SARS-CoV-2 after the epidemic—older, higher rate of cardiac involvement, had features of Macrophage activation syndrome and associated with a 30 times higher incidence of a severe form of Kawasaki disease.		Small case series. Kawasaki like disease—rare condtion (0.001 children affected by SARS-CoV-2)
	Group 2: *n* = 10				
Antona et al.	156	France	Kawasaki-like disease (61%), myocarditis (70%), macrophage activation syndrome (23%), seritis (22%)	67% with one death vasopressors−73%	
Zahra Belhadjer et al.	35	France and Switzerland	Comorbid—asthma and overweight	64% with no death	
			Complication—Acute cardiac decompensation. Left ventricular systolic function recovered with immunoglobulin.	29%—Invasive mechanical ventilatory support	
Marie Pouletty et al.	16	France	Hemodynamic failure, Orchitis, Aseptic meningitis, Raynaud syndrome and Anosmia	44% with all in remission.	Direct link between the Kawasaki Disease and SARS-CoV-2 not demonstrated.
			Respiratory features observed in adult COVID patients were not seen.		
Maria Paz Deza Leon et al.	1	Europe	Female, 6 years old	Admitted in PICU	Case report
			Underlying group A Streptococcus infection		
			Treatment—IVIG, aspirin, ECMO		
			COVID-19 milder in children—a genetic predisposition for cardiac complications or a previously unrecognized inflammatory response to COVID-19.		
Astrid Elisabeth Rojahn et al.	1	Norway	Comorbid—Food allergies.	Transferred to PICU with cardiogenic shock, Incipient multiorgan failure, hypotension, oliguria, altered sensorium, and tachypnea.	Case report
			Increase in incidence of the disease after 3–4 weeks of COVID-19 peak suggests a delayed immune response.		
Antonio Torrelo et al.	4	Spain	Target and targetoid skin lesions, confluent macules, papules and plaques, with different sizes, some with hemorrhage or a small central crust.		Case series
Kim et al.	768	Korea	The incidence of Kawasaki Disease in Korea is 217.2 per 100,000 children < 5 years old, 10–30-fold higher than that of KD in North America and Europe.		Editorial
Toubiana et al.	21	France	Myocarditis, Kawasaki like shock syndrome, coronary artery dilatations, GIT symptoms.	81% with no deaths	Small sample size
			High proportion of the affected children and adolescents were of African ancestry		
Shelly Riphagen et al.	8	UK	Males dominant	100% with 7/8 requiring mechanical ventilation. Discharged after 4–6 days. 1 death.	Small sample size
			Warm, vasoplegic shock, refractory to volume resuscitation—treated with noradrenaline and milrinone.		
			Adenovirus and enterovirus were isolated.		
Kathleen Chiotos et al.	6	UK	Females dominant.	100% with 3/6 intubated and 2/6 non-invasive mechanical ventilation.	Small sample size
			Myocardial dysfunction, troponin leak, severe enteropathy and relative thrombocytopenia.	1/6 stayed in PICU, others discharged after 8–17 days.	
			67%—neurological symptoms		
			Patient 4—aseptic meningitis consistent with Kawasaki disease.		
Ramcharan et al.	15	UK	Male dominan	100% with deaths. Discharged on aspirin.	Small case series, unable to establish management—treatment guidelines and some patients not referred.
			Treatment—Norepinephrine and vasopressin, Epinephrine.		
			Impaired left ventricular function, valve regurgitation and/or coronary artery involvement, systemic hypotension.		
Mike Masih et al.	1	UK	Male, 9-year-old		Case report
			History of asthma.		
			PMIS-TS is a post infective, delayed antibody-mediated dysregulated immune response, with an onset between 2 and 4 weeks after initial infection.		
Michele et al.	1	UK	Male, 11 years old	Admitted in PICU, requiring high-flow nasal cannula support (15 liters per minute, 50% FiO2)	Case Report
			Comorbid—Pneumonia		
			High-grade conduction system disease is a potential complication of MIS-C.		
Swann et al.	651	UK	Male dominant	18% with 9% requiring mechanical ventilation	Case record form as data collection.
			Asthma, neurological problems, immunocompromised, premature births, hematological and oncological co-morbids, obesity.		Initially, diagnostic serology was not available.
			Children who met the WHO preliminary definition for MIS-C were significantly older, of non-white ethnicity and five times more likely to be admitted to critical care and receive mechanical ventilation.		Loss of follow up.
Patrick Davies et al.	78	UK	Males' dominant	100%	
			High proportion of Asian and Afro-Caribbean children	Mechanical ventilation−36, ECMO−3	
Dolinger et al.	1	USA	Male, 14-year-old	Admitted	Case report
			Comborbids—Crohn's disease		
			Treated with infliximab for TNF-α blockade.		
Andrea et al.	1	USA	Female	Admitted	Case report
			Treated with enoxaparin injections		
			MIS-C affects children beyond infancy		
Nele Alders et al.	57	UK	Comorbids—Overweight/Obese	63% with 37% mechanically ventilated.	Incomplete data due to referral nature of the center.
					Small, localized sample size.
Feldstein et al.	186	USA	The 4 patients who died were 10–16 years of age; 2 of the patients had diagnoses of underlying conditions.	80% with 20% mechanically ventilated. 3/186 received ECMO support.	Results are not generalizable.
				4 deaths.	No comparison group.
					Retrospective chart.
Dufort et al.	99	USA	Males' dominant	80% with 2 deaths	Initially, limited availability of testing.
			Comorbid—Obesity	Mechanical ventilation−10%	
Kaushik et al.	33	USA	Males' dominant.	67% with 1 death.	
			Comorbids—overweight/obese	Mechanical ventilation−5	
Bandi et al.	474	USA	Male dominant	12%, 1 intubated	Small sample size
			Asthma		Type 2 error in assessing risk of COVID-19 in asthmatic patients
			No ED or hospital admissions for children with asthma—asthma not a risk factor for COVID-19 in children nor a severe disease.		
Cheung et al.	17	USA	Females' dominant	88% with 59% on vasoactive support.	Small sample size
					Short follow up period
					Inability to establish causality
Chiu et al.	1	USA	Male, 10-year-old		Case report
			Stable vital signs and a normal ambulatory saturation.		
			Severely diminished left ventricular systolic function with trace pericardial effusion.		
Maria et al.	1	USA	Female, 6 years old	Admitted to PICU, ECMO started	Case report
			Syncope on day 3 of illness. Maculopapular rash on all extremities. Prominent cardiac silhouette and mildly decreased left ventricular function.		
Einat Blumfield et al.	16	USA	Males dominan	69% with 1 on mechanical ventilation	Small sample size
			Comorbids—Obesity, asthma, sickle cell disease, ventricular septal defect and UTI.		
			In children with MIS-C associated with COVID-19, the most common thoracic imaging abnormalities were cardiomegaly, congestive heart failure or pulmonary edema, and pleural effusions.		
Heidemann et. al	3	USA	Presented with vasculitis and cardiac manifestations who responded to intravenous immunoglobulin and aspirin.	1 admitted, 2 intubated.	Small sample size
Mariawy Riollano-Cruz et al.	15	USA	Males' dominant	93% with 53% mechanically ventilated	
			Comorbids—Asthma, Hypothyroidism, non-alcoholic fatty liver disease, respiratory illness	1 death—required ECMO during the 9 days of admission.	
			Treated with broad spectrum antibiotics and prophylactic anticoagulation with Enoxaparin	One patient required an intra-aortic balloon pump to treat cardiogenic shock.	
			The disproportionate burden of disease among Hispanic/Latino and black/African–American ancestry		
DeBiasi et. al	177	USA	Male dominan		Retrospective design.
			Comorbid—Asthma, Neurologic, Diabetes, Obesity, Cardiac, Hematologic and Oncologic		
Shanana Godfred et al.	570	USA	Male dominant	63.9% with 10 deaths.	Possibility of reporting bias.
			Comorbid—Obesity	Intubated−13%	Inconsistency in completion of case report forms.
			Long-standing inequities in housing, economic instability, insurance status, and work circumstances of patients and their family members have systematically placed social, racial, and ethnic minority populations at higher risk for COVID-19 and MIS-C.		
Shema Hameed et al.	35	USA	Males dominant	68.5% with one death due to extensive right cerebral infarct whilst on ECMO.	Small sample size
				Mechanical ventilation−20%	
Rivera-Figueroa et al.	1	USA	Male, 5 years old	Admitted with high flow nasal cannula. Discharged after 6 days.	Case Report
Elaine et al.	1	Brazil	Male, 10 years old		
			Discharged at 14th day of hospitalization		
Omar Yassef et, al	409	Latin America	Male dominant		Some cases misdiagnosed as no confirmatory test and that the CDC case definition is broad.
			Pre-existing medical condition, known immunodeficiency, respiratory tract infection, gastrointestinal symptoms and low socio-economic conditions were associated with PICU admission		
Al-Aamria et al.	1	KSA	Female, 10–15 years old	Admitted, intubated and ventilated	Case report
			FST for G6PD screening was positive	Died at day 33 due to multiple organ dysfunction syndrome.	
Daniel et al.	1	USA	Male, 14 years old	Admitted, intubated and mechanical ventilation	Case report
			Comorbid—constipation and eczem	Discharged at 12-day on low dose aspirin and penicillin G prophylaxis.	
			Family history for ulcerative colitis		
**MISC in upper-middle and low-and middle-income countries (1)**					
Hançerli Törün et al.	570	Turkey	Comorbid—obesity and Chronic Lung Disease	63.9%	
			Cardiovascular involvement—most common clinical characteristic (493)		
Ozsurekci et al.	52	Turkey	Comorbid—neurometabolic/genetic disorders, hematologic/oncologic and chronic pulmonary disease		Retrospective study with a small sample size
			No deaths in MIS-C group		
Haslak et al.	76	Turkey	Kawasaki disease, cardiac murmur, hepatomegaly and musculoskeletal findings	27 (35.5%)	
Shafique et al.	8	Pakistan	Fever (for more than 3 days), stomachache, vomiting, diarrhea, red eyes, rashes on the trunk and shock.		Lack of awareness among clinicians
					Restricted of access to healthcare
					Poor referral system
Bahrami et al.	1	Iran	History of upper respiratory symptoms over the past 3 weeks.		Case report
			At the time of discharge—evidence of desquamation in fingers was observed. Prescribed low dose aspirin (3 mg/kg daily) and repeat echocardiogram after 1 week.		
Arnaldo Prata Barbosa et al.	79 (13% had MIS-C)	Brazil	Males' dominant	Mechanical ventilation−14, Discharged−90%	Results are not generalizable.
			Comorbid—Non-progressive encephalopathy, chronic respiratory disease, onco-hematological disease, congenital heart disease, under nutrition.		Some lacking details about treatment and investigations.
			ARDS−71%. No deaths in MIS-C group.		
Satareh Mamishi et al.	45	Iran	Mortality−11%		
			Comorbids—acute lymphocytic leukemia, chronic kidney disease, cerebral palsy and Budd– Chiari syndrome		
			Clinical presentation predominantly consisted of sepsis-like disease and toxic-shock like disease		
Kate Webb et. al	23	South Africa	Males' dominant.	52% due to cardiac abnormalities. No deaths.	Patients were not tested for COVID-19
			Comorbids—Pre-natal HIV exposure, Obesity, AML, Epilepsy		
Balasubramanian et al.	1	India	Male, 8-year-old	Admitted, recovered in 2 weeks.	
			MISC shares common features with KD, Staphylococcal/streptococcal toxic shock, bacterial sepsis and macrophage activation syndrome.		
			High CRP levels—mediated by IL-6.		
Torres et al.	27	Chile	Comorbid—overweight, asthma, primary immunodeficiency, GATA 3 deficiency, prematurity and gestational age of 33 weeks	59% with duration of stay of 5 days.	Small sample size
				O2 support−13/27	Lack of definitive outcome.
				Mechanical ventilation−12/27	Loss of follow up.
					Chances of underreporting.

### MIS-C and socio-economic factors

Given the ethnically diverse nature of the aforementioned HICs, the high number of cases suggests a relationship between socio-economic factors and MIS-C. Higher COVID-19 infection rates have been associated with lack of insurance, overcrowded neighborhoods where social distancing is ineffective, and high exposure jobs within the service industry, transport, and healthcare sectors which are dominated by people of color (62). Especially Hispanic families who mostly live in metropolitan areas in apartment buildings, bigger families, and mainly use public transport (63). This can lead to adults exposing more children to coronavirus at home and serve as a possible explanation for the increased number of COVID-19 cases progressing to MIS-C. Discrimination within the healthcare system, limited healthcare access because of lack of transportation to take their children to the hospital on time, cultural and linguistic barriers, inability to take time off work, possible distrust in the system due to inherent biases and fears of deportation for symptomatic adults also play a role in acquiring timely access to healthcare (64, 65). A retrospective case-control study in Massachusetts conducted on 44 patients with MIS-C (Hispanic = 44%, Black = 26%) concluded that a higher social vulnerability index (SVI), lower socio-economic status (SES), Hispanic ethnicity, and Black race were independently associated with developing MIS-C (66). Mitigating social determinants of health is important as future winter waves of SARS-CoV-2 are anticipated. Improved housing decreases in overcrowding, and improved nutrition has for years proven to be effective interventions for controlling respiratory infections such as tuberculosis (67). Factors such as reducing smoke exposure, financial support to low-income households, improving access to healthcare, free and accessible testing, and provision of shelter to those in need have great potential to improve future pandemic morbidity and mortality (68).

## Conclusion

This review highlights the need for high-quality data on ethnicities and socio-economic positions of patients affected by MISC, especially in regions severely impacted by COVID-19. Social determinants of health should be routinely considered in clinical assessments the same way as age and sex, as they can play an important role by aiding in the creation of tailor-made policies for risk mitigation. It is important to note the equitable distribution of resources, such as critical care and hospital beds for pediatric patients with MISC, is essential for reducing mortality because most of the resources were allocated to adult COVID-19 patients. In LMICs, where lockdown policies put vulnerable populations such as the elderly and children at a higher risk of exposure as most people live in overcrowded conditions, there should be strict surveillance. Good standard healthcare is not free in most LMICs, hospitals funded by the government are found to be stretched, and the cost associated with prolonged hospital admissions and critical care can be a factor that holds back families from seeking hospital care until an emergency arises. In addition to social determinants of health, comorbidities were another driving factor leading to the overrepresentation of ethnic minorities getting affected by MISC. Further genetic studies are also needed to warrant the role of genetic susceptibility to MISC in children. This review can form the basis of larger cohort studies investigating the role of ethnicity and social determinants of health in developing MISC that pose a serious public health concern. Our review also elucidates the importance of cross-cultural prospective cohorts to correctly assess the wide clinical variability of this syndrome and help us solidify common socio-economic and racial/ethnic factors driving the severity of MIS-C. Including data from LMICs helped in gaining a new perspective for the occurrence of this syndrome as we saw how delays in attaining appropriate treatment, unavailability of critical care and a lack of timely diagnosis led to severe forms of disease. In conclusion our review identified similar patterns of racial findings, socio-economic strata and limitations of health set ups across different countries of the same economic classification which can aid policy makers in making effective strategies to mitigate the development of MIS-C therefore further studies in the same area should be focused on.

## Author contributions

ZA conceived the idea, developed the methodology for the study, and wrote the first draft. ZA, KS, FB, OS, and MS were equally involved in literature review, synthesizing results from the literature, and writing and editing of the manuscript. AW contributed to editing. IU and AN contributed in the overall structure and editing of the manuscript. All authors read, critically analyzed, edited, and approved the final manuscript.

## Conflict of interest

Author AN is employed by Hamad Medical Corporation. The remaining authors declare that the research was conducted in the absence of any commercial or financial relationships that could be construed as a potential conflict of interest.

## Publisher's note

All claims expressed in this article are solely those of the authors and do not necessarily represent those of their affiliated organizations, or those of the publisher, the editors and the reviewers. Any product that may be evaluated in this article, or claim that may be made by its manufacturer, is not guaranteed or endorsed by the publisher.
